# Wanted: A population genetic theory of biological noise regulation

**DOI:** 10.1371/journal.pgen.1012066

**Published:** 2026-03-13

**Authors:** Daniel M. Weinreich, Tom Sgouros, Yevgeniy Raynes, Hlib Burtsev, Edison Chang, Sanyu Rajakumar, Ignacio G. Bravo, Csenge Petak

**Affiliations:** 1 Department of Ecology, Evolution and Organismal Biology, Brown University, Providence, Rhode Island United States of America; 2 Data Science Institute, Brown University, Providence, Rhode Island United States of America; 3 Laboratory MIVEGEC (Univ Montpellier, CNRS, IRD) Centre National de la Recherche Scientifique, Montpellier France; 4 Department of Biology, University of Vermont, Burlington, Vermont United States of America; University of Rochester, UNITED STATES OF AMERICA

## Abstract

Classical population genetics provides a robust, quantitative framework for modeling how natural selection acts on alleles that influence phenotypes with invariant fitness consequences for their carriers, such as running speed or drug resistance. By contrast, modifier theory considers the evolution of alleles that influence population genetic parameter values in their carriers, such as mutation or recombination rates. This is a more complicated problem. First, the fitness effects of modifier alleles reflect independently realized stochastic phenotype perturbations they induce in their carriers. And second, the association between modifier alleles and their induced phenotypes can decay over generations. Consequently, general results in modifier theory have been few. Here, we propose recasting modifier theory as exploring the evolution of alleles that influence the amount of stochasticity in inheritance, be it genetic, epigenetic, cytoplasmic or somatic transmission. We then present a toy model that predicts the existence of a selectively optimal amount of such “reproductive noise,” which depends on the rate of environment change, the timescale of association between noise allele and induced phenotype, and population size. Next, we suggest that the same framework can be applied to the evolution of alleles that influence “developmental noise,” i.e., the amount of stochastic phenotypic variation among genetically identical organisms reared in identical environments. This theoretical connection is timely, because high throughput assays are now demonstrating widespread heritability in the amount of developmental noise. Our approach also resolves the long-standing teleological criticism of the hypothesis that evolvability can evolve by natural selection. Taken together, this work demonstrates the opportunities for a robust, quantitative population genetic theory of alleles that influence the amount of biological noise.

## Author summary

Darwin’s theory of evolution describes how mean trait values change in response to natural selection over generations. But if mean trait values are subject to natural selection, what about the amount of variation (or noise) around those means? This is more than an academic question: genetic variation introduced between generations is the fuel for evolution, including the evolution of therapy resistance in pathogenic microbes and agricultural pests. And trait variation introduced within generations is essential for developmental flexibility and plasticity, but is also the basis of adaptive immunity and underlies some disease. Extending established work on the evolution of population genetic parameters like mutation and recombination rates, we propose a unified framework for studying the evolution of the amount of all sources of biological noise, whether introduced between or within generations. We present a toy model that captures both the costs and opportunities introduced by biological noise, and which suggests the existence of a selectively optimal amount of noise. Recent technological advances in our ability to characterize many sources of biological noise make this work timely, and we conclude by suggesting steps for extending and generalizing our ideas.

## Introduction

Population genetics asks how a population’s genetic composition changes over generations in response to mutation, recombination, migration, natural selection and random genetic drift. Classical textbook models of natural selection assume that, holding genetic, environmental, social, ecological and all other features constant, a given allele has a fixed phenotypic (and thus, fitness) effect on its carriers. Under this assumption, the evolutionary fate of an allele can be computed by recursively applying this fitness consequence to every descendant carrier in which it appears [1]. This is called “direct selection” because it assumes that an allele’s identity directly determines its selective effect [2,3].

Modifier theory [4–6] is usually defined as the study of alleles that modify population genetic parameter values in their carriers, such as their mutation or recombination rates. The phenotypic and fitness consequences of a modifier allele often vary among carriers, since they can induce distinct genetic effects in each one. Selection on modifier alleles is thus also mediated by statistical associations with directly selected alleles elsewhere in the genome, and this is called “indirect” [2,3,7] or “second order” [8,9] selection.

Notably, modifier alleles are not unique in their sensitivity to indirect selection. For example, statistical associations mediated by genetic linkage mean that directly selected beneficial or deleterious alleles at one locus will indirectly influence the frequency of selectively neutral alleles at nearby loci. (These effects are called genetic hitchhiking [10] and background selection [11], respectively.) And models for the evolution of cooperation [12–14] explicitly partition the fitness of an allele for altruistic behavior into a direct effect (typically a cost) and an indirect effect, mediated by the carrier behavior’s (typically beneficial) influence on the fitness of socially associated individuals also carrying the allele.

Importantly, modifier alleles can simultaneously be subject to indirect and direct selection. For example, fitness in some RNA viruses depends in part on genome replication rate [15,16]. Thus, if an RNA polymerase allele lacking a proofreading activity replicates the genome faster, it will increase fitness in all carriers. Such direct selection is independent of any indirect selection mediated by the phenotypic consequences of whatever mutations the error-prone polymerase introduces.

Even putting aside the complication of direct selection acting on modifier alleles, the associations driving their indirect selection can decay over time, as for example due to genetic recombination between a mutation rate modifier and whatever directly selected mutation(s) it has induced. This decay further complicates modifier theory, and direct attacks on the general problem have been quite technical (e.g., [17,18]). Indeed, we know the most about the evolution of mutation rate modifiers in asexual organisms (e.g., [19–21]), precisely because the association between such modifiers and resulting phenotypes is permanent.

## Recasting modifier theory as the evolution of reproductive or between-generation noise regulation

**Fig 1:**
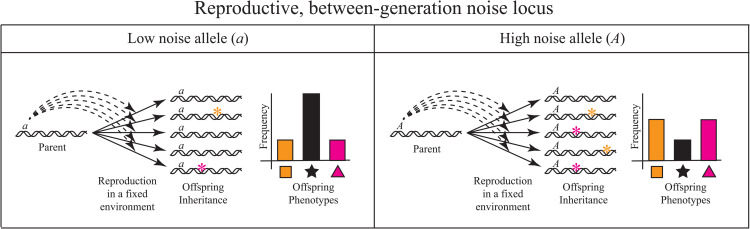
Genetic regulation of reproductive, between-generation noise. Holding parent and environment constant, low and high reproductive noise modifier alleles (designated *a* and *A*) differentially influence the amount of variation among offspring inheritances (represented by colored asterisks) and thus, among offspring phenotypes (represented by colored shapes). Note that the double helices represent the totality of the inheritance, including genetic, epigenetic, cytoplasmic, and somatic components.

Altenberg [22] took another approach, suggesting that modifier theory be regarded as the study of alleles that influence the fidelity of genetic transmission. Here, we extend that idea to include stochasticity in all aspects of inheritance, be it genetic, epigenetic, cytoplasmic or somatic transmission. We designate such stochasticity as reproductive (i.e., between-generation) noise. Fig 1 schematically illustrates the influence of low and high reproductive noise modifier alleles on phenotypic variability in the offspring of the same parent, and Table 1 enumerates many biological mechanisms that influence reproductive noise.

**Table 1:** Biological mechanisms that influence reproductive, between-generation noise.

Mechanism	Consequence	Comments	Representative citations
Genetic mutation	Increasing mutation rate increases genetic variation among offspring.	DNA polymerase alleles can also vary in mutational bias, e.g., [23–26], sometimes with evolutionary consequences [27].	[3]
Mode of reproduction	Sexual reproduction introduces genetic variation among offspring absent in asexual or clonal reproduction.	We distinguish between modifiers of mode of reproduction and modifiers of recombination rate (next).	[28]
Genetic recombination	Increasing genetic recombination rate increases genetic variation among gametes.	Unlike modifiers of sexual reproduction (previous), modifiers of genetic recombination rate influence the probability of recombination between loci, which can occur in haploids as well as in obligate sexual diploids.	[17,29]
Mendelian segregation distortion	Segregation distorters (meiotic drivers) bias allele transmission and reduce genetic variation among gametes.	Complicated by the fact that these alleles can also be directly selected. Bias in genetically determined sex ratio [2,30] is a special case of segregation distortion.	[31]
Heritable DNA and histone modification	Reducing fidelity of DNA or histone modification over organismal generations increase epigenetic variation among offspring.		[32]
Asymmetry in cytoplasmic inheritance	Increasing asymmetry in cytoplasmic inheritance increases variation in cytoplasmic content of daughter cells (e.g., mitochondria number).	It is perhaps formally interesting to notice that increasing asymmetry here increases heterogeneity among daughter cells, whereas in the case of genetic segregation distortion (above), increasing asymmetry reduces noise. In the present case, transmission is zero-sum: one daughter cell inherits whatever the other does not. Whereas with genetic segregation, asymmetry enriches the population for the driver allele at the expense of the other allele.	[33]
Fidelity in other forms of non-genetic inheritance	Increasing fidelity of vertical transmission of cytoplasmic and somatic features of an organism (including the microbiomes of plants and animals) reduces variation among offspring.		[34–36]
Ploidy	Higher ploidy buffers the phenotypic effects of recessive deleterious mutations, thus diminishing phenotypic diversity.	In this sense, increasing ploidy is analogous to lowering the deleterious mutation rate. Higher ploidy can also increase the recombination rate.	[37,38]
Proportion of each generation spent in the haploid phase	Increasing the proportion of time spent in the haploid phase increases the phenotypic consequences of new, recessive mutations.	In this sense, increasing the proportion of time spent in the haploid phase is analogous to raising the mutation rate.	[39]
Assortative mating	Increasing the rate of assortative mating reduces genetic variation among offspring by increasing homozygosity.		[40]

**Fig 2:**
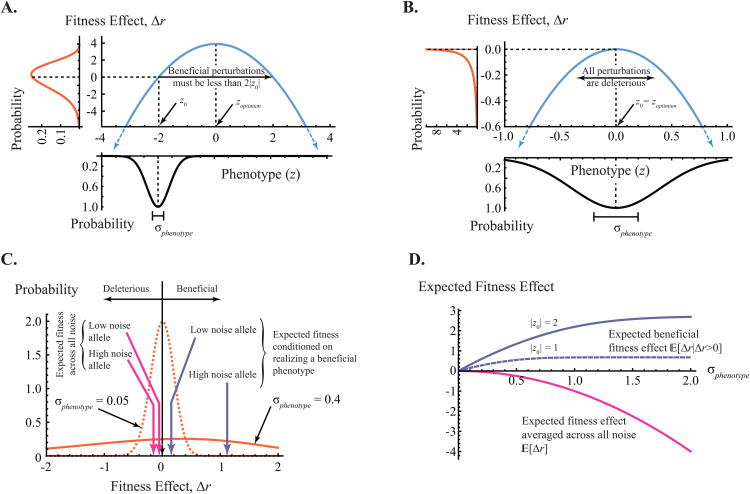
Fitness effects of phenotypic noise under toy model introduced in text. **A.** First, we assume normally distributed noise in a single phenotype *z* (Eq. 1), shown in black below the x-axis for expected value $z_{0}=𝔼[z]=-2$ and magnitude $\sigma_{phenotype}=0.4$. Next, we assume a quadratic phenotype-to-fitness function, shown in blue above the x-axis. Without loss of generality, we set the phenotypic fitness optimum $z_{optimum}=0$ (Eq. 2). The resulting probability density function of fitness effects Δr (Eq. 3) is shown in orange to the left of the y-axis. Because $z_{0}\neq z_{optimum}$, some phenotypic perturbations are beneficial. **B.** All phenotypic perturbations are deleterious if an organism’s expected phenotype is at the fitness optimum ($z_{0}=𝔼[z]=z_{optimum}=0$). $\sigma_{phenotype}=0.4$ as in panel A, though the x-axis has been rescaled for emphasis. **C**. In the absence of direct selection (i.e., holding $z_{0}$ constant), probability density functions for fitness effects induced by high and low noise modifier alleles, shown respectively by solid orange ($\sigma_{phenotype}=0.4$, as in left curve of panel A, though rescaled for emphasis) and dotted orange ($\sigma_{phenotype}=0.05$). Averaged over all phenotypes, the higher the noise, the lower the expected fitness effect (compare the two magenta arrows). But when averaging only over the ensemble of phenotypes with positive fitness effects, the higher the noise, the higher the expected fitness effects (compare the two violet arrows). **D.** Magenta: expected fitness effect averaged over all phenotypes as a function of the magnitude of noise $\sigma_{phenotype}$ (Eq. 4). This is independent of the expected phenotype $z_{0}$. Violet: expected fitness averaged over (or conditioned on) the ensemble of beneficial phenotypes as a function of $\sigma_{phenotype}$ (Eq. 5). Shown for expected phenotype $|z_{0}|=2$ (solid, as in panels A and **C)** $|z_{0}|=1$ (dashed).

Recasting modifier theory as the study of alleles that influence the amount of reproductive noise admits a simple yet suggestive toy model for their evolution. By definition, the phenotypic consequences of reproductive noise are random. Correspondingly, we present the stochastic model shown in Fig 2 to illustrate how random phenotypic perturbations influence an organism’s fitness. Firstly, we assume normally distributed perturbations of a one-dimensional phenotype (e.g., [41]) represented by the random variable *Z*. Algebraically,


$$Z\sim N(z_{0},\sigma_{phenotype}^{2}),\;$$ (1)

where $\sigma_{phenotype}$ is our proxy for the magnitude of the noise and $z_{0}$ is the organism’s expected phenotype averaged over noise (formally, $z_{0}=𝔼[Z]$). Eq. 1 is shown in black, below the x-axis of Fig 2A and B, and we consider more complex forms of phenotypic noise in the Discussion.

Following others (e.g., [41,42]), we next assume that Malthusian fitness (*r*) drops quadratically in realized *z* from an optimal phenotype ($z_{optimum}$), written $r(z)=r_{optimum}-(z_{optimum}-z{)}^{2}$. Without loss of generality, we set the optimal phenotype $z_{optimum}=0$. Thus, the fitness effect of a phenotypic perturbation to *z* realized in an organism with expected phenotype $z_{0}$ is


$$\Delta r(z,z_{0})=r(z)-r(z_{0})=z_{0}^{2}-z^{2},$$ (2)

shown in blue above the x-axis of Fig 2A and B.

Finally, the change-of-variable technique (Methods) yields an expression for the probability density function of fitness effects (Eq. 3) for given expected phenotype (*z*_0_) and magnitude of noise (*σ*_*phenotype*_). Typical results are shown in orange to the left of the y-axis in Fig 2A and B, and above the x-axis in Fig 2C.

As detailed next, our model predicts the existence of a selectively optimal amount of reproductive noise, regardless of mechanism (Table 1). To simplify, we follow others (e.g., [5,7]) and assume that modifier alleles only experience indirect selection, which in our notation means holding *z*_0_ constant. The interested reader is directed to [2] and [43], where this assumption is relaxed.

## Reproductive noise is deleterious on average

In our model, all phenotypic perturbations larger than $2|z_{0}|$ are deleterious (Fig 2A), and since we assume that noise is symmetric in *z*, only half the remainder are beneficial. Moreover, for a given phenotypic perturbation smaller than $2|z_{0}|$, deleterious outcomes will be more deleterious than beneficial outcomes are beneficial. This effect, called Jensen’s inequality [44], follows because fitness effect Δr (Eq. 2) is concave down. Thus on expectation over all phenotypes, phenotypic noise is deleterious (Fig 2C; algebraically, $𝔼[\Delta r(z,z_{0})]<\Delta r(𝔼([z],z_{0})=0$.)). Indeed under our model, expected fitness goes down without limit as reproductive noise goes up (Eq. 4, Methods; shown in magenta in Fig 2D). Interestingly, Eq. 4 is independent of $z_{0}$; somewhat surprisingly, it is also independent of Eq. 1 (see Eq. 4a).

The limiting case is an organism at the phenotypic fitness optimum ($z_{0}=𝔼[z]=z_{optimum}=0$). Here, all phenotypic perturbations are deleterious (Fig 2B). In this case, natural selection will always favor low-noise alleles. Indeed, even in populations only at equilibrium between natural selection, mutation, recombination, migration and random genetic drift (so, not quite at the phenotypic optimum), natural selection will favor low-noise mutation, migration, or recombination modifier alleles. This is the reduction principle [18], one of the few general results we know from modifier theory.

## Reproductive noise can sometimes be beneficial

But while the unconditioned fitness effect of reproductive noise goes down as noise goes up, in general (i.e., if $z_{0}\neq z_{optimum}$), increased noise also increases access to high fitness phenotypes (Fig 2C). Indeed, conditioned on realizing a beneficial phenotype, expected fitness goes up with reproductive noise before reaching a plateau under our model (Eq. 5, Methods; shown in violet in Fig 2D for two values of $z_{0}$, illustrating that the plateau goes up as $\left|z_{0}-z_{optimum}\right|$ goes up).

We suggest that the tension between these unconditioned (Eq. 4, algebraically $𝔼[\Delta r(z,z_{0})]$) and conditioned (Eq. 5, $𝔼[\Delta r(z,z_{0})|\Delta r(z,z_{0})>0]$) fitness expectations (respectively the magenta and violet lines in Fig 2D) is critical to understanding the evolutionary fate of reproductive noise modifier alleles. In particular, the rates of change in the slopes of these two quantities (their second derivatives) differ under our model. The unconditioned fitness expectation (magenta) drops faster as noise goes up, while the conditioned fitness expectation (violet) goes up more slowly as noise goes up.

Together, this suggests the existence of a selectively optimal amount of reproductive noise. From there, higher noise alleles will be selectively disfavored, because their now more modest conditioned fitness advantage is likely to be offset by their greater unconditioned fitness cost. And at the same time, lower noise alleles will also be selectively disfavored, because their reduced ability to capitalize on anomalously high fitness phenotypic realization is not offset by their reduced unconditioned fitness cost. We suggest three factors that may influence the location of this selective optimum.

1. The selectively optimal amount of reproductive noise depends on the rate of environmental change

Under our model, the scope for fitness improvement induced by reproductive noise goes down as an organism’s expected phenotype $z_{0}$ approaches the phenotypic fitness optimum $z_{optimum}=0$ (compare solid and dashed violet lines in Fig 2D). Now relaxing the assumption that the environment (and hence, the fitness function) is invariant in time, an evolving population will lag behind its moving fitness optimum in phenotypic space by an amount that goes up with the rate of environmental change. (See [41,45] for early theoretical work, and [46] for a more recent empirical application.) Thus, when such change is directional, we predict that the selectively optimal amount of biological noise similarly goes up with the rate of environmental change (although this reasoning breaks down under some models of cyclically varying environments, e.g., [41]).

2. The selectively optimal amount of reproductive noise depends on the timescale of association between noise allele and induced phenotype

The location of the selectively optimal amount of reproductive noise must also depend on the timescale (in generations) of association between noise alleles and their resulting phenotypic perturbations. This follows because indirect selection can only distinguish between segregating noise alleles in a population via whatever directly selected phenotypic differences they happen to have recently induced in their carriers. Put another way, indirect selection’s efficacy will depend on the correlation between parent and offspring phenotype.

One limit is represented by mutation rate modifier alleles in non-recombining organisms. These remain permanently associated with whatever phenotypic perturbations they cause via mutations elsewhere in the genome. Because phenotypic correlation between parent and offspring is thus maximized, indirect selection can reliably distinguish between competing noise alleles. This is why high noise mutation rate alleles (known as mutators) often reach fixation in asexual microbial populations challenged by novel lab environments [19,47–51]. It also explains why low noise mutation rate alleles (known as antimutators) often reach fixation in asexual populations evolving in an environment to which they are already well adapted [20,47,50,52,53], though see [54]. We note that these two results are also consistent with our first prediction: that the selectively optimal amount of biological noise should be positively correlated with the pace of environmental change.

Intermediate timescales of association are represented by mutation rate modifiers in sexual organisms. In laboratory experiments, such mutators struggle to succeed even when challenged by a novel environment [49], since indirect selection only transiently favors them before they are decoupled by recombination from whatever beneficial phenotype they may induce. Carja and Plotkin [55] treat the general case using simulations, confirming that a high noise allele’s probability of success goes up with timescale of association with induced phenotype. Interestingly, this effect will plateau, since the time to fixation of a directly selected mutation scales with the reciprocal of its fitness advantage [56]. Thus, indirect selection’s capacity to favor high noise alleles is probably maximized as soon as timescales of phenotypic associations are of that order.

Finally, Gillespie [57] treats the other limit: high noise alleles almost never succeed when parent and offspring phenotypes are entirely uncorrelated. This follows because while still rare, the risk that the high noise allele is lost in the next generation as a consequence of sampling a deleterious phenotype is of much greater evolutionary significance than is the (one-generation) benefit of increased frequency as a consequence of its sampling a beneficial one.

3. The selectively optimal amount of reproductive noise depends on population size

It has long been recognized that the strength of random genetic drift scales inversely with population size, or equivalently, that as population size goes up, natural selection can reliably detect ever smaller fitness differences [30,58]. For a population at its phenotypic fitness optimum, all noise is deleterious (Fig 2B), meaning that in such populations, low noise alleles are always favored. We thus predict that in populations at the phenotypic fitness optimum, the amount of noise should be inversely correlated with population size. This is Lynch’s “drift barrier” hypothesis [59], which is consistent with the pattern observed for mutation rates across cellular life (*r*^2^ ≈ 0.8, [60]).

However the picture is more complicated in populations not at the phenotypic fitness optimum, since some stochastic phenotypic fluctuations are now beneficial (Fig 2A and C). The strength of random genetic drift of course still goes down as population size goes up. And assuming that most phenotypic perturbations are deleterious, indirect selection against high noise alleles will be the first effect to overcome drift [61]. Thus in populations of moderate size, we still predict a drift barrier effect (i.e., selection against the high noise allele).

But as population size continues to increase, the strength of indirect selection favoring high noise alleles will eventually come to exceed both the strength of random genetic drift and indirect selection against them [61]. Thus, we predict that at above some critical population size, the selectively optimal amount of noise will go up with population size. This non-monotonicity in the direction of selection acting on noise alleles is called “sign inversion” [21]. It emerges from the double-edged nature of reproductive noise, and has been observed in several systems [21,37,57,62,63]. We speculate all reproductive noise alleles will exhibit sign inversion in populations not at their phenotypic fitness optimum.

## A selectively optimal amount of developmental or within-generation noise

**Fig 3:**
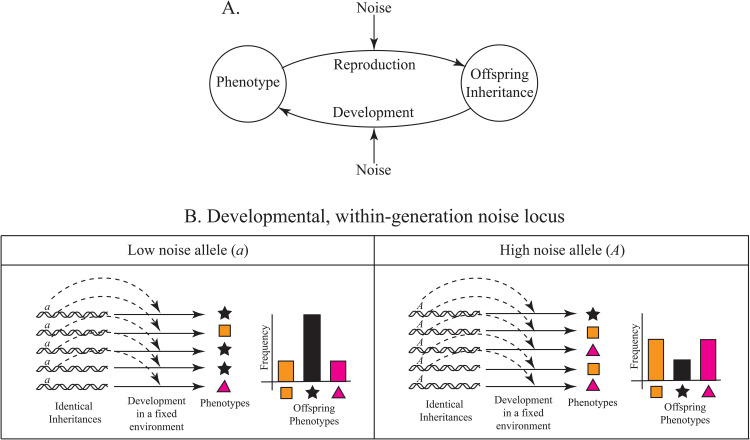
Generalized theory applies equally to the genetic regulation of developmental, within-generation noise. **A.** During reproduction (upper arrow) information flows from the parent to the next generation’s inheritance and during development (lower arrow), it flows from the offspring’s inheritance to its phenotype. This implies that biological noise can emerge between or within generations, suggesting two classes of genetic loci that influence its amount. **B.** Holding inheritance and environment constant, low and high developmental noise modifier alleles (designated *a* and *A*) differentially influence the amount of phenotypic variation (represented by colored shapes) among organisms. For illustrative purposes, we assume a single, scalar phenotype. Note that the double helices again represent the totality of the organism’s inheritance, including genetic, epigenetic, cytoplasmic and somatic components. Fig 1 presents the analogous image for reproductive noise modifier alleles.

Biological information flows both between and within generations (Fig 3A, [64]). This suggests that the foregoing theory may apply equally to alleles that influence the amount of noise introduced during development. Here, we use the word development in its broadest sense, to include all aspects of molecular biology, biochemistry, metabolism, cell biology, biophysics, physiology, behavior, life history, as well as the traditional domains of developmental biology.

Fig 3B schematically illustrates the influence of low and high developmental noise alleles on phenotypic variation among offspring endowed with otherwise identical inheritance and reared in identical environments. This last point is critical. Previous work by Elowitz and colleagues [65] has demonstrated the difference between “extrinsic” and “intrinsic” noise in gene regulation. The former refers to phenotypic perturbations induced by stochastic environmental perturbations, while the latter are phenotypic perturbations above and beyond environmental noise. As Elowitz et al [65] explain, these latter can emerge from “random microscopic events… arising from the discrete nature of the biochemical process of gene expression.” And recent evidence demonstrates that intrinsic effects can influence organismal behavior [66]. Here, we are largely concerned with the evolution of alleles that influence intrinsic noise, and Table 2 enumerates some biological mechanisms that give rise to this kind of noise.

**Table 2:** Biological mechanisms that influence developmental, within-generation noise.

Mechanism	Consequence	Comments	Representative citations
Gene expression noise	Increasing variation in RNA or protein abundance over time or space increases phenotypic variation among genetically identical cells.		[67–69]
RNA polymerase and ribosome noise	Increasing variation in protein sequence over time or space increases phenotypic variation among genetically identical cells.	Includes mis-transcription, post-transcriptional modification variation, tRNA-charging errors, mis-translation and post-translational modification variation.	[70,71]
Enzyme promiscuity	Increasing variation in substrate binding affinity and/or catalytic rate increases phenotypic variation among genetically identical cells.	We follow [72] and restrict the term to enzymes that also catalyze physiologically irrelevant secondary reactions. We thus distinguish between promiscuous enzymes and those with broad, physiologically important substrate specificity, e.g., cytochrome P450 enzymes. (Note too that structural proteins can exhibit binding promiscuity [73,74].)	[72]
Protein fold-switching	Increasing propensity for protein fold-switching increases heterogeneity in protein structure over time or space among genetically identical cells.	Also called metamorphic proteins [75] or extant fold-switching [76], this effect is closely related to evolved fold-switching (e.g., [77]), in which alternative fold structures come to be separated by only very few mutations.	[78]
Regulation, signaling, metabolic or ecological network topology	Network topology can influence variation in network behavior among genetically identical cells.		[79,80]
Environmental canalization	Increased environmental canalization reduces environmentally triggered phenotypic variation among genetically identical organisms.	Also called “environmental robustness”. Often in opposition to diversified bet hedging.	[81]
Phenotypic plasticity	Increased phenotypic plasticity increases phenotypic variation among genetically identical organisms in different environments. (It may be favored by indirect selection if it reduces mismatches between phenotype and variable environments.)	Following others (e.g., [82]), we restrict phenotypic plasticity to cases where environmental cues trigger specific phenotypic responses, e.g., as described by norms of reaction. We thus distinguish phenotypic plasticity from random phenotypic perturbations such as diversified bet hedging (see previous).	[83]
Seasonal migration	Increased seasonal migration reduces variation in environmental conditions encountered by genetically identical organisms.	Modifiers of dispersal (next) increase variation in environment experienced, while modifiers of seasonal migration reduce variation in environment experienced.	[84]
Dispersal	Increasing dispersal increases variation in environment experienced by otherwise identical offspring.	If modeled as a per-generation probability of migration to a different environment *m*, an organism’s environment is heritable for 1/*m* generations in expectation. In contrast, modifiers of seasonal migration (previous) act within generations.	[85]
Distribution of fitness effects or DFE	Modifiers of the DFE influence variation in selection coefficients acting on the same mutation in otherwise identical organisms.	These include modifiers of mutational robustness, genetic (de)canalization, and evolutionary capacitors. This class of modifier loci can cause populations to systematically move across the fitness landscape in response to indirect selection, i.e., to cause the local fitness landscape experienced by an evolving population to evolve (e.g., [86] but see also [87]). Complicated by the fact that these alleles are also intrinsically directly selected.	[43]
Epistasis	Modifiers of epistasis influence variation in phenotypic effects of an allele at one locus across alleles at other loci in the genome.	Complicated by the fact that these alleles are also intrinsically directly selected.	[88]
Dominance	Modifiers of dominance influence the variation in phenotypic effects of an allele at one locus across alleles at the homologous locus in diploids and polyploids.	Complicated by the fact that these alleles are also intrinsically directly selected.	[2,89]
Pleiotropy	Modifiers of pleiotropy influence the variation in multiple phenotypic consequences of a single mutation.		[90,91]
Genetic assimilation and the Baldwin effect	Increasing capacity for genetically encoding beneficial developmental and learned behavioral phenotypes reduces phenotypic and behavioral variation among offspring.		[92]

The most obvious formal difference between developmental and reproductive noise modifier alleles lies in the lower heritability of phenotypes induced by developmental noise. For example, consider a locus in a single-celled organism that influences the variance in cellular concentration of some biomolecule. Now imagine an individual in which a high noise allele has induced a selectively favorable, anomalously high (or low) concentration of that molecule. While the high noise allele may then enjoy an immediate indirect selective advantage, degradation and dilution of the biomolecule will quickly push such perturbations back toward their expectations.

Nevertheless, while indirect selection on developmental noise alleles may be weakened by their shorter associations with beneficial phenotypes, recall that any parent-offspring correlation in phenotype is sufficient for the effect [55]. Consistent with that result, there is empirical support for a weak drift-barrier signal among developmental noise alleles that influence single-point transcription [93,94] and splicing [95] error rates. And transgenerational phenotypic plasticity persists for several generations after the original environmental cue (reviewed in [96]). Thus, indirect selection may find reliable signals with which to distinguish between competing plasticity noise alleles. The observation of sign inversion in modifiers of a canonical form of developmental noise (bet hedging) [97–99] also suggests that developmental noise alleles respond to indirect selection [61].

Another critical distinction between reproductive and developmental noise is that the former happens exactly once per generation, while the latter often induces phenotypic perturbations more frequently. This is perhaps especially important in long-lived organisms with distinct developmental phases. Moreover, the distributions from which developmental perturbations are sampled can themselves vary over an organism’s lifetime (e.g., [100,101]). Similarly, in multicellular organisms developmental noise can be sampled from different distributions among cells [102] or cell types [103].

In summary, we believe that our toy model applies equally to reproductive and developmental noise modifier alleles, despite their mechanistic differences. It will be of interest to see whether more complex models also predict selectively optimal amounts of both reproductive and developmental noise, determined by the physico-chemical and spatio-temporal details of the noise, the timescales of environmental change, and the population size.

## Discussion

### Modifier theory and the evolution of evolvability

Recasting modifier theory as the population genetics of biological noise regulation has an important implication for understanding the evolution of evolvability, defined as a organism’s capacity to generate heritably adaptive variants [104–107]. The distinction between evolvability-as-byproduct and evolvability-as-adaptation [106,108] in that literature is equivalent to distinguishing between direct and indirect selection acting on evolvability alleles. Evolvability-as-adaptation arguments have been criticized as teleological, since an evolving population cannot “anticipate” what phenotypes are likely to be beneficial in the future (e.g., [83,106,109,110]). Reframing the evolution of evolvability in terms of biological noise regulation appears to resolve this concern, since noise is inherently a double-edged sword. As we have suggested, indirect selection increases biological noise only when increasing the population’s capacity to generate adaptive phenotypic variants (its evolvability) outweighs the concomitant cost of generating deleterious variants. Otherwise, indirect selection will reduce biological noise because this reduction in the population’s capacity to generate deleterious phenotypes (sometimes called its robustness) now outweighs the possibility of generating adaptive phenotypes.

### Next steps

We suggest that modifier theory be understood as the population genetics of alleles that heritably influence the amount of reproductive or developmental noise, and our toy model predicts the existence of a selectively optimal amount of both classes of biological noise. We now plan to quantitatively locate that optimum as a function of the three determinants enumerated above using individual-based simulations. We are hopeful that that work will in turn lead to a mathematical solution.

Of course, our model makes several strong assumptions. For example, in contrast to Eq. 1, noise modifier alleles might influence more than the second moment of the phenotypic distribution. They can also simultaneously change distributions in several traits, as when they influence the pleiotropic effects of mutations [90,91,111,112]. And we assumed a Gaussian fitness function (Eq. 2) simply out of mathematical convenience, but other models are possible. Even non-continuous models for the fitness function are recognized, such as truncation selection, which emerges in several real-life biological situations (reviewed in [113,114]). Interestingly, truncation selection reduces the fitness penalty induced by high noise alleles, increasing their chances of evolutionary success [113].

Moreover, while it appears reasonable to assume that the fitness function is concave down near the phenotypic optimum, the local topography can change as the function moves away from that value, as for example if the fitness function asymptotically decreases towards a minimum or if it is multimodal. This suggests that after a sufficiently large environmental perturbation, a population might find itself in a concave-up region of the fitness function, in which case Jensen’s inequality implies that indirect selection would always favor high-noise alleles. On the other hand, as natural selection drives such a population back toward the (local) fitness optimum, our earlier predictions will once again apply.

Ultimately, our prediction of a selectively optimal amount of biological noise rests on an empirical question: does the fitness cost of noise increase in an accelerating manner with noise while its fitness benefit rises in a decelerating manner? We thus look forward to the development of datasets exploring the distribution of fitness effects of biological noise for specific mechanisms and processes across biological systems and conditions. Tables 1 and 2 provide a list of candidates, and we hope that questions raised here will motivate others to ask whether the unconditioned and conditioned expectations of the resulting distributions exhibit the same trends as those of our toy model. More abstractly, it will be of interest to see whether any model features are shared among underlying mechanism. And finally, do more realistic models admit quantitative solutions for the selectively optimal amount of biological noise?

## Conclusions

The population genetics of alleles that influence mean trait values have been well established for almost a century. Here we propose that modifier theory be recast as the population genetics of alleles that influence the amount of stochastic variation around those means: the amount of biological noise. This generalization unites several previously disparate domains of theoretical inquiry and is timely because technological advances are revolutionizing empirical access to phenotypic stochasticity across time scales and levels of biological organization (Table 1 and 2). Biological noise also has important implications for human disease, and the evolution of therapy resistance of pathogenic microbes and in cancer (e.g., [102,115]). We are optimistic that the ideas introduced here will lead to an integration of experimental approaches, models and theory resulting in a deeper understanding of origin and evolution of the forces responsible for observed levels of biological noise in nature.

## Methods

If random phenotype $Z\sim N(z_{0},\sigma^{2})$ (Eq. 1; we suppress the subscript on *σ* for convenience) and fitness effect $\Delta r=z_{0}^{2}-z^{2}$ (Eq. 2; we suppress the arguments on Δr for convenience), the change-of-variable technique (shown here) gives the probability density function for fitness effects Δr:


$$f_{\Delta r}(\Delta r;z_{0},\sigma )=\frac{{\text{e}}^{-\frac{(-\sqrt{z_{0}^{2}-\Delta r}-z_{0}{)}^{2}}{2\sigma^{2}}}+{\text{e}}^{-\frac{(\sqrt{z_{0}^{2}-\Delta r}-z_{0}{)}^{2}}{2\sigma^{2}}}}{2\sqrt{2\pi }\sigma \sqrt{|-\Delta r+z_{0}^{2}|}}.$$ (3)

This in turn yields the expected fitness effect over all phenotypic perturbations:


$$𝔼[\Delta r]\equiv \int_{-\infty }^{r(0,z_{0})}f_{\Delta r}(\Delta r;z_{0},\sigma )\Delta r\text{d}\Delta r=-\sigma^{2}.$$ (4)

Recall that without loss of generality we set *z*_optimum_ = 0, making *r*(0,*z*_0_) the appropriate upper limit of integration. Dirk Metzler (*pers. comm.*) pointed out that the form of Eq. 2 yields an even more direct path to Eq. 4:


$$𝔼[\Delta r]=𝔼[z_{0}^{2}-z^{2}]=𝔼[𝔼[z{]}^{2}]-𝔼[z^{2}]=-(𝔼[z^{2}]-𝔼[z{]}^{2})\equiv -\sigma^{2}.$$ (4a)

Thus, this result is independent of the probability density function of phenotypes (Eq. 1).

Finally, the expected fitness effect conditioned on a beneficial phenotype reads


$$\;𝔼[\Delta r|\Delta r>0]\equiv \frac{\int_{0}^{r(0,z_{0})}f_{\Delta r}(\Delta r;z_{0},\sigma )\Delta r\text{d}\Delta r}{\int_{0}^{r(0,z_{0})}f_{\Delta r}(\Delta r,z_{0},\sigma )\text{d}\Delta r}\;=\frac{2|z_{0}|\sqrt{\frac{2}{\pi }}\sigma +(z_{0}^{2}+\sigma^{2})\left(\text{Erf}\left[\frac{z_{0}-|z_{0}|}{\sqrt{2}\sigma }\right]-\text{Erf}\left[\frac{z_{0}+|z_{0}|}{\sqrt{2}\sigma }\right]\right)}{\text{Erf}\left[\frac{|z_{0}|\sqrt{2}}{\sigma }\right]}+z_{0}^{2}.$$ (5)

All derivations were performed in Mathematica, as was the production of Fig 2. That code is available here under GNU General Public License (GPLv3), and can be examined with the free Wolfram Player application. Eqs. 3–5 match simulation results developed in MatLab (not shown).
